# Reflective Functioning in Children and Adolescents With and Without an Anxiety Disorder

**DOI:** 10.3389/fpsyg.2021.698654

**Published:** 2021-09-20

**Authors:** Valérie Chevalier, Valérie Simard, Julie Achim, Pamela Burmester, Thalie Beaulieu-Tremblay

**Affiliations:** ^1^Department of Psychology, Université de Sherbrooke, Sherbrooke, QC, Canada; ^2^Research Center of the Sainte-Justine University Hospital, Montreal, QC, Canada

**Keywords:** reflective functioning, anxiety, internalizing symptoms, children, adolescents, mothers, attachment, mentalization

## Abstract

Reflective functioning (RF), meaning the capacity to interpret mental states (intentions, emotions, thoughts, desires, and beliefs) underlying one’s own and others’ behaviors, may help understand the dysfunctional self-regulation associated with anxiety disorders. However, research on anxiety and RF in clinical samples is scarce. This study aimed to assess whether mothers’ and youths’ RF was associated with youths’ (a) anxiety disorders and symptoms and (b) internalizing symptoms. Another goal was to explore whether RF predicted anxiety and internalizing symptoms beyond the more commonly established effect of attachment. Canadian children and adolescents aged between 8 and 16years, and their mothers were recruited in an outpatient psychiatric clinic (clinical group with a diagnosed anxiety disorder, *n*=30, mean age=11.5±2.8years) and in the general population (non-clinical group, *n*=23, mean age=11.5±2.1years). The Child Attachment Interview was used to assess youths’ attachment along with three dimensions of RF (global, regarding self, regarding others). Mothers’ attachment and RF were assessed with the Adult Attachment Interview. Children’s and adolescents’ anxiety and internalizing symptoms were measured with the Behavior Assessment Scale for Children, second version. The clinical and non-clinical groups did not differ in mothers’ or youths’ RF. However, in the overall sample, youths’ RF regarding themselves and maternal attachment preoccupation were associated with internalizing symptoms. Sequential regression analyses revealed that higher RF regarding self predicted a higher level of self-reported internalizing symptoms, beyond the effect of maternal attachment (*β*=0.43, *p*<0.05). This study’s finding suggests that clinically anxious children and adolescents have adequate RF. We propose that the sustained hypervigilance and apprehension associated with anxiety make anxious youths sensitive to their own and others’ mental states. Our findings suggest that psychotherapeutic treatments for anxiety should make use of patients’ RF abilities to help them make sense of their symptoms and thus reduce them.

## Introduction

Anxiety disorders affect 6.5% of school-aged children and adolescents, making it the most prevalent class of mental disorders in this age group (Polanczyk et al., 2015). Moreover, their lifetime prevalence is as high as 15–20% (Beesdo-Baum and Knappe, 2012) and they are among the most persistent mental disorders (Kessler et al., 2012). They show high rates of homotypic (anxiety disorders) or heterotypic (other disorders) comorbidities (Beesdo-Baum and Knappe, 2012). Specifically, the comorbidity between anxiety and depression among children and adolescents has been widely documented (e.g., Stein et al., 2001; Brückl et al., 2007; Beesdo et al., 2010), with reported rates being as high as 30% (Essau, 2003). Although both conditions may be conceptualized as nosological entities (i.e., disorders or diagnoses), they are also defined as symptomatologies in the broader spectrum of internalizing difficulties, which refers to behavioral, social, and emotional problems related to anxiety, depression, and somatization (American Psychiatric Association, 2013; Achenbach et al., 2016). The present study assesses children’s and adolescents’ anxiety from both the medical (anxiety disorders) and dimensional (anxiety and broader internalizing problems) perspectives in relation to mothers’ and youths’ psychological characteristics likely to affect emotion regulation.

Attachment theory postulates that, from early childhood, anxiety arises from attachment insecurity, i.e., one’s implicit prediction and lack of confidence that others will be available or responsive when needed (Bowlby, 1969). More recently, attachment was conceptualized as the “central organizer” of the risk factors for the development of the dysfunctional self-regulatory processes underlying anxiety disorders (Nolte et al., 2011). Nevertheless, studies on child attachment and anxiety have yielded inconsistent findings; some found associations between the two (Colonnesi et al., 2011; Kerns and Brumariu, 2014), while others did not (Groh et al., 2012). This has led some authors to stress the importance for future research not only to assess *if* attachment is related to anxiety, but *why* (Kerns and Brumariu, 2014). We propose that reflective functioning (RF), an intrinsically relational variable closely linked to the ability to regulate affects (Fonagy and Target, 1998), would help better understand how attachment is linked to anxiety. Specifically, we hypothesize that RF, which develops in the context of the parent–child relation, would explain variance in anxiety beyond the effect of attachment.

RF is considered as the empirical operationalization of mentalization, i.e., the capacity to interpret mental states (intentions, emotions, thoughts, desires, and beliefs) underlying one’s own and others’ behaviors, making them meaningful and predictable (Fonagy et al., 1991, 2002; Slade, 2005). High RF capacities are characterized by efforts to tease out the mental states’ underlying behaviors and by the awareness of mental states’ nature (e.g., their opaqueness, potentiality to be disguised, and interdependence; Fonagy et al., 1998). Although breakdowns in mentalization are common in contexts of emotional overload and of acute triggering of the attachment system (Midgley et al., 2017; Luyten and Fonagy, 2019), persistent difficulties have mostly been associated with personality disorders (e.g., Fonagy et al., 2002; Bateman and Fonagy, 2004). Nevertheless, failures in mentalizing are also thought to be present in a broad range of psychopathologies encountered by clinicians in psychotherapy with adults (Fonagy et al., 2012), but also in children (Midgley et al., 2017; Achim et al., 2020a). Therefore, mentalization-based treatments have gained popularity in all types of clinical settings.

In the last decade, there has been a growing interest in how RF and anxiety are related. For instance, it has been suggested that parents’ anxiety, attachment insecurity, and low RF would altogether influence their capacities to understand and discuss their children’s emotional states, likely leading to dysregulation and anxiety (Esbjørn et al., 2012). Similarly, dysfunctional emotion regulation in the attachment relationship (i.e., exaggeration or inhibition of distress expression in response to a threat, such as separation) is thought to impede the child’s RF development, which would subsequently contribute to the development of anxiety (Nolte et al., 2011). It has also been suggested that features of anxiety such as emotional arousal, social abilities deficits, and hypervigilance toward the environment would be associated with RF difficulties (Midgley et al., 2017). However, empirical research on the association between RF and the internalizing difficulties spectrum remains scarce. Some studies have shown that low RF was associated with internalizing problems among adolescents (Badoud et al., 2015; Duval et al., 2018), while others reported the opposite association (high RF associated with more severe anxiety symptoms; Chow et al., 2017). These conflicting findings may reflect underlying fluctuations in attachment, as most measures of RF explicitly or implicitly trigger the attachment system. Indeed, given that RF develops in the context of the attachment relationships (Fonagy and Target, 1998), it is reasonable to expect an impact of one’s attachment representations on their RF capacities. Therefore, in the present study, the relationship between RF and anxiety will be examined after controlling for attachment.

As previously suggested, the association between RF and anxiety may also vary as a function of the specific facets of mentalization being assessed (Breinholst et al., 2018). RF is indeed a multidimensional construct (Fonagy and Bateman, 2019). Based on works in the neuroscience of social cognition (Lieberman, 2007; Luyten and Fonagy, 2015), four distinct dimensions are at play in the mentalization process: automatic vs. controlled; self-oriented vs. others-oriented; internal vs. external; cognitive vs. affective (Fonagy and Bateman, 2019). The self-oriented vs. other-oriented dimension is arguably the most commonly studied in the developmental psychopathology field (e.g., Ensink et al., 2014; Borelli et al., 2017). RF regarding self (RF-Self) refers to the capacity to recognize, identify, and understand one’s own mental states, while RF regarding others (RF-Others) is the ability to mentalize the behaviors, emotions and thoughts of others (Luyten et al., 2019). RF-Self and RF-Others would have distinct patterns of associations with psychosocial adjustment (Luyten and Fonagy, 2015). For instance, a study in a psychiatric inpatient sample of adolescents revealed that internalizing symptoms were negatively associated with RF-Self but not with RF-Others (Borelli et al., 2017). With respect to anxiety specifically, these distinct dimensions of RF remain to be studied. It could be hypothesized that, as for internalizing difficulties, RF-Self could pose a bigger challenge for anxious children and adolescents considering the emotional self-regulation difficulties associated with anxiety (Mathews et al., 2016). Moreover, previous studies suggest that anxious children would be fairly good at recognizing others’ mental states due to their tendency to constantly analyze their environment (Ale et al., 2010). Thus, the limited pool of current studies seems to point toward contradictory results. Being able to define the specific RF capacities of anxious youths could contribute to more accurate therapeutic interventions.

Finally, given the well-documented contribution of parenting variables in the development and maintenance of anxiety (Kertz and Woodruff-Borden, 2011; Nolte et al., 2011; Yap and Jorm, 2015), it is feasible to assume that parents’ RF is associated with youth’s anxiety. However, the association between parents’ RF and children’s mental health also remains unclear. Low RF was found among mothers of psychiatric outpatient children (Dubois-Comtois et al., 2019), and low RF in mothers, coupled with high attachment avoidance in fathers, was linked to anxiety symptoms reaching the clinical level in children (Esbjørn et al., 2013). To our knowledge, no study has assessed parents’ and youths’ RF in association with anxiety, conceptualized either as a diagnosis or in terms of symptoms, or with the broader internalizing difficulties spectrum.

**Figure 1:**
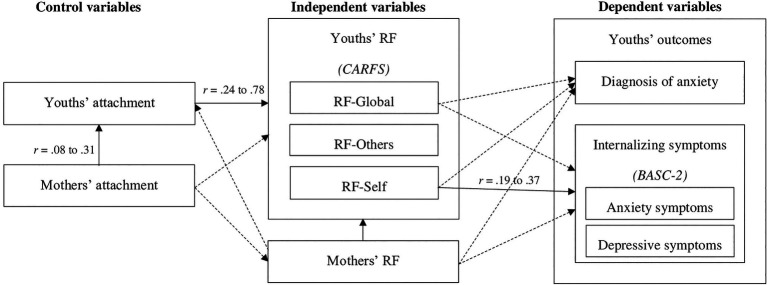
Theoretical and observed bivariate associations between the study variables. Dotted lines represent theoretical associations that were not significant in the present study. Operationalizations are shown in brackets. All direct associations between RF and youths’ outcomes are expected to be negative. RF=Reflective functioning; CARFS=Child and Adolescent Reflective Functioning Scale; RFS-AAI=Reflective functioning scale for application to the AAI; CAI=Child Attachment interview; AAI=Adult Attachment interview; BASC-2=Behavior Assessment Scale for children, second version.

This study’s general objective was to assess the relative contributions of mothers’ and youths’ RF to child and adolescent anxiety (disorder and symptoms) and internalizing difficulties (anxious and depressive symptoms), while controlling for attachment. Given the well-documented association between attachment and anxiety and the fact that RF is thought to develop within the attachment relationship, we explored whether RF predicts anxiety and internalizing symptoms beyond the effect of attachment. This study also aimed to explore the specific contributions of different dimensions of RF (mothers’ general RF and youths’ general, self, and other-related RF) to children’s and adolescents’ anxiety disorders and internalizing difficulties (anxious and depressive symptoms). Based on the theoretical models and preliminary empirical evidence presented above, we hypothesized that lower levels of mothers’ general RF and youths’ general RF and RF-Self (but not RF-Others) would predict more anxiety and internalizing symptoms, as well as the presence of a diagnosed anxiety disorder (Figure 1).

## Materials and Methods

### Participants

#### Clinical Group

As part of a larger study, 30 children and adolescents (18 girls) with a diagnosed anxiety disorder (DSM-IV criteria; American Psychiatric Association, 2000) and their mothers (*n*=30) were recruited by psychiatrists of an outpatient clinic specializing in the assessment and treatment of anxiety disorders at Sainte-Justine University Hospital (Montreal, Canada). To participate in the study, youths had to be aged between 8 and 16years (*M*=11years 5months, *SD*=2years 10months). Exclusion criteria were to have a primary diagnosis of post-traumatic stress or obsessive–compulsive disorders, which are no longer classified as anxiety disorders in the DSM-5 (American Psychiatric Association, 2013). Children and adolescents with comorbid conditions other than anxiety disorders were included, as long as anxiety was the primary diagnosis according to the psychiatric assessment. In addition to the psychiatric assessment, a structured diagnostic interview was administered by graduate psychology students [Schedule for Affective Disorders and Schizophrenia for School-Age Children-Present and Lifetime version (K-SADS-PL); Kaufman et al., 1997]. According to the K-SADS-PL’s assessment of anxiety disorders, 46.7% (*n*=14) of youths had specific phobia, 33.3% (*n*=10) had generalized anxiety disorder, 26.7% (*n*=8) had panic disorder (with or without agoraphobia), 26.7% (*n*=8) had separation anxiety disorder, 10% (*n*=3) had social phobia, and 10% (*n*=3) had a non-specified anxiety disorder. Most youths had one anxiety disorder (53.3%, *n*=16), whereas one-third of the sample (33.3%, *n*=10) had two concurrent anxiety diagnoses and 13.3% had three (*n*=4). Although this rate of homotypic comorbidity (46.7%) is slightly higher than reported in the non-clinical population (e.g., Canals et al., 2019), it is reasonable to assume higher comorbidity in an outpatient clinic specializing in the treatment of anxiety disorders.

#### Comparison Group

A non-clinical sample composed of 23 healthy children and adolescents (16 boys), also aged between 8 and 16 years (*M*=11years 6months, *SD*=2years 1month), and their mothers (*n*=21) were recruited through social media and word of mouth. The inclusion criteria for the comparison group were as follows: (a) child/adolescent with no history of a diagnosed mental or neurological disorder and (b) child/adolescent not currently engaged in psychotherapy or taking psychoactive medication.

The overall sample (*n*=53) is composed of middle-class families. The clinical and comparison did not differ in family income, maternal education and youths’ age. In both groups, yearly family income was in the 80,000 to 100,000 CAD range, which corresponds to the median family income of the province of Québec (98,690 CAD; Statistique Canada, 2021) where families were recruited. Mothers’ education level was equivalent in both groups. Nearly half of mothers (46.6%) had at least a university degree, which is higher than the proportion in the general population of the province of Québec (25.5%; Statistique Canada, 2017). There was, however, a difference between the groups in the gender ratio [*X*^2^(1, *N*=53)=4.57, *p*=0.03] girls representing 60% of the clinical group and 30% of the comparison group. This distribution is representative of the higher prevalence of anxiety disorders in girls than in boys (approximately 2:1 ratio; American Psychiatric Association, 2013).

### Procedure

Upon reception of their contact information, families were first contacted by a research assistant, who provided detailed information on the study objectives and procedures, inquired about the inclusion and exclusion criteria, and planned a home (clinical group) or in-laboratory (comparison group) visit. Two trained graduate psychology students administered the Adult Attachment Interview (AAI) to mothers and the Child Attachment Interview (CAI) to youths, in separate rooms. To enable their verbatim transcription and subsequent scoring, the AAIs were audio recorded and the CAIs were video recorded. In both groups, the Behavior Assessment Scale for Children, second version (BASC-2), was completed in the days following the visit and returned by mail in a pre-addressed and prepaid envelope. This resulted in a reduced sample size in the clinical group for the analyses using the BASC-2 (23 out of 30 dyads).

This research project received full approval by the scientific and ethical boards of the University of Sherbrooke and Sainte-Justine University Hopistal (Canada).

### Materials

#### Adult Attachment Interview

The AAI (George et al., 1984, 1985, 1996) is a semi-structured interview that approximately takes 1h to administer and consists of 20 open-ended questions and follow-up probes. The questions elicit the participants’ reflections on their childhood experiences with their attachment figures, and the lasting effects of these experiences through adulthood. The AAI is considered the “gold standard” to assess adult attachment representations and is also the main measure used with the Reflective Functioning Scale (Fonagy et al., 1998). The AAI’s attachment scoring system (Main et al., 2002) has been widely used, and its psychometric properties are well-established (for a review, see Hesse, 2016). Scoring is done through discourse analysis by an independent coder that provides scores on several 1-to-9 state-of-mind scales and assigns the transcript to one of four attachment classification (secure–autonomous, insecure–dismissing, insecure–preoccupied, or unresolved). Given the relatively modest sample size and low prevalence of insecurity in the present study, analyses were conducted using a dimensional – rather than categorical – approach to attachment. To do so, we computed composite scores based on the AAI alternative two-factor model of Haltigan et al. (2014). The dismissing factor included the scores of the “coherence of mind,” “idealization of father/mother,” and “defensive lack of memory” scales, whereas the preoccupation factor included scores of the “coherence of mind,” “preoccupying anger toward father/mother,” “passivity in discourse,” and “unresolved trauma” scales. All the transcripts (*n*=51) were coded by VS (trained by Sonia Gojman de Millan), and interrater reliability was established with another certified coder (trained by June Sroufe and Sonia Gojman de Millan) on 54.7% (*n*=29/53) of transcripts. The interrater agreement was excellent for all the AAI scales used in the computation of dimensional scores (ICC from 0.75 to 0.93), except for the “idealization of father” scale for which the agreement was good (ICC=0.66).

##### Reflective Functioning Scale

The Reflective Functioning Scale for application to Adult Attachment Interviews – 5th edition (Fonagy et al., 1998), was used to assess mothers’ RF. The scoring system is based on four dimensions of RF: “awareness of the nature of mental states,” “efforts to tease out mental states underlying behavior,” “recognizing developmental aspects of mental states,” and “showing awareness of mental states in relation to the interviewee.” RF is assessed based on the participant’s answers to specific AAI “demand questions,” i.e., questions demanding to think about the feelings and intentions behind their attachment figures’ behaviors (e.g., Why did your parents behave as they did during your childhood?). Each of these specific passages is scored on a−1 to 9 scale. A score of −1 represents hostility toward the process of RF, a score of 0 is given in the absence of RF, and scores from 1 to 9 represent minimal to exceptional RF, with a score of 5 considered “good” RF. In addition to those “demand questions” scores, every other AAI question is considered a “permit question,” that is, one where the participant *can* but does not *have to* demonstrate some reflective capacity (e.g., What did you do when you were upset as a child?). Those passages are not given a specific RF score but are considered in the attribution of the global RF score in the overall interview rating. The RF scale applied to the AAI has good interrater reliability (Fonagy et al., 1998) and is not associated with mood state, self-esteem, personality (extraversion, neuroticism, psychoticism), or intelligence (Fonagy et al., 1998; Steele and Steele, 2008). All transcripts were coded by VC (trained by Howard Steele), and 20% of the transcripts (*n*=10/51) were double coded by another certified coder (TB-T, trained by Howard Steele). Both coders were blind to the participants’ group (clinical vs. comparison) and attachment scores. Interrater agreement was excellent for the global RF score and each demand question (ICC=from 0.87 to 0.97), except for the question on closeness with attachment figures for which the agreement was good (ICC=0.74).

#### Child Attachment Interview

Youths’ RF and attachment were assessed with the CAI (Target et al., 2007), which is an adaptation of the AAI for children and adolescents. This 30- to 45-min semi-structured interview aims to activate the attachment system by asking questions about relational episodes and moments of vulnerability (e.g., illness and separation) involving the attachment figures. Unlike the AAI, the CAI taps into youths’ current relationships with their parents and assesses attachment to mother and father separately. As for the AAI, the participant’s discourse is rated on several 1- to 9-point Likert scales and attachment classifications (to each parent) are attributed based on the profile of scores across these scales and on the discourse’s general characteristics (Shmueli-Goetz et al., 2011). The CAI’s attachment coding system shows good psychometric properties (Privizzini, 2017). As for the AAI, we used a dimensional approach to youths’ attachment to retrieve, once again, as much relevant attachment information considering the small sample size and the uneven distribution of attachment classifications in our sample. Composite scores derived from the CAI two-factor model (Zachrisson et al., 2011) were computed from the standardized scores on the attachment interview scales relevant to each factor. The preoccupation–idealization factor includes the “preoccupied anger” and “idealization of attachment figures” scales, and the security–dismissing factor includes the “emotional openness,” “balance of positive/negative references to attachment figures,” “use of examples,” “resolution of conflicts,” and “idealization of attachment figures” scales. All the transcripts (*n*=53) were coded by VS (trained by Yael Shmueli-Goetz), and 33% of the transcripts of the clinical group (n=10/30) were double coded by another certified coder (also trained by Yael Shmueli-Goetz). Interrater agreement was excellent for all scales used to compute the attachment dimensions’ scores (ICC from 0.75 to 0.99). Both coders were blind to the mothers’ attachment representations when scoring youths’ attachment.

##### Child and Adolescent Reflective Functioning Scale

The Child and Adolescent Reflective Functioning Scale (CARFS; Ensink et al., 2015) is the RF scale for application to the CAI for children and adolescents aged from 8 to 17years. The same four dimensions of RF (i.e., “awareness of the nature of mental states,” “efforts to tease out mental states underlying behavior,” “recognizing developmental aspects of mental states,” and “showing awareness of mental states in relation to the interviewee”) assessed in the adult system are adapted to suit children’s and adolescents’ cognitive, affective, and social development levels. Similar to the RF scale for the AAI, the CARFS assesses RF based on the participant’s response to specific questions, that is, those where children are asked to describe (a) themselves, (b) relationships with their attachment figures, (c) conflicts with them, (d) conflicts between their parents, and (e) situations when they felt upset. A principal component analysis of the CARFS (Ensink, 2004) and a subsequent validation study (Ensink et al., 2014) confirmed that RF-Self and RF-Other stand as distinct dimensions that can be reliably assessed with this coding system. Scores of RF-Self and RF-Others are derived from questions specifically eliciting those themes (Self: “description of self,” “self-upset”; Others: “relationship with mom/dad,” “mom/dad angry,” “parental conflict”). Moreover, a global RF score is given to the interview based on the whole transcript, including passages that were not specifically rated for RF. The CARFS shows good interrater reliability, stability over a 3-month period, and discriminant validity related to child abuse and trauma (Ensink, 2004; Ensink et al., 2014). All transcripts of the non-clinical sample were coded by VC, and transcripts of the clinical sample were coded by another certified rater (PB), both trained by Ensink. Raters were blind to the youths’ attachment but were provided with their age and clinical status. Interrater reliability was established on 27% (n=8/30) of the clinical sample’s transcripts and 26% (n=6/23) of the non-clinical sample’s transcripts. Interrater agreement was excellent for all scales (ICC from 0.88 to 0.98).

#### Behavior Assessment Scale for Children, Second Version

The Behavior Assessment Scale for Children, second version (BASC-2; Reynolds and Kamphaus, 2004), is a multi-informants system of questionnaires that assesses adaptive functioning and problematic behaviors from age 2 to 25 years. It was used in the present study to assess anxiety and internalizing problems in the child or adolescent with the Self-Report of Personality (SRP), and the Parent Rating Scales (PRS), completed by the mother. The SRP for children aged 8 to 11 (139 items) and that for adolescents aged 12 to 21 (176 items) were used in this study, along with the PRS for parents of children aged 6 to 11 (160 items) and for parents of adolescents aged 12 to 21 (150 items). Items are to be answered in True/False and four-point Likert-scale (*Never* to *Almost always*) response formats. The Internalizing Problems scale includes scores from the Anxiety, Depression, and, only in the adolescents’ version, Somatization subscales. The Internalizing Problems and Anxiety scales of the SRP and PRS show good-to-excellent internal consistency (Cronbach’s α from 0.80 to 0.95). The SRP and PRS also have shown adequate to excellent test–retest reliability over a 20- to 45-day interval for the Internalizing Problems (0.82 to 0.93) and Anxiety (0.70 to 0.86) scales. To avoid controlling for age in the regression models, all analyses were conducted using the BASC’s standardized (*t*) scores.

### Data Analyses

Preliminary analyses were conducted using independent samples *t* tests to look at differences between the clinical and non-clinical groups on the main study variables (attachment and RF scores), and zero-order correlations were performed to look at the associations between anxiety and internalizing symptoms and the study variables. Based on these preliminary analyses, we further investigated the predictive effect of different dimensions of RF on youths’ symptoms, with and without controlling for relevant covariates (variables associated with youths’ symptoms). Specifically, multiple linear regressions predicting youths’ anxiety and internalizing symptoms were first performed with RF-Self and RF-Others as independent variables and no control variables. To assess RF’s predictive effect beyond the effect of attachment, sequential regressions were performed with youths’ symptoms as dependent variables and RF-Self and RF-Others as independent variables, both with and without controlling for youths’ gender. The regression models included no multivariate outlier according to Mahalanobis distance. The visual inspection of the standardized residuals plot revealed that the assumptions of normality, linearity, and homoscedasticity were met. The data were analyzed using IBM SPSS Statistics Version 26 for Mac OS.

## Results

### Preliminary Analyses

As displayed in Table 1, youths’ and mothers’ attachment and RF scores did not differ between the clinical and non-clinical groups. Because these groups did not differ in the main study variables, no further analyses were conducted to investigate the predictive role of RF on the presence of an anxiety disorder. Furthermore, a dependent *t* test revealed that the overall sample’s score of RF-Others (*M*=4.13, *SD*=1.38) was significantly higher than the score of RF-Self [*M*=3.52, *SD*=1.04; *t*(52)=−3.79, *p*=0.000]. Mothers’ RF in the overall sample (*M*=4.17, *SD*=1.90) was slightly below the “ordinary RF” threshold score of 5 on the RF scale.

**Table 1:** Descriptive statistics and between-group differences in the main study variables.

	Overall sample (*N*=53)		A Clinically anxious (*n*=30)		B Non-anxious (*n*=23)		A vs. B	
	Mean	SD	Mean	SD	Mean	SD	*t* value	Cohen’s *d*
Sociodemographics								
Youths’ age	11.50	2.48	11.47	2.78	11.55	2.06	0.11	0.03
Youths’ RF								
RF-Global	4.17	1.63	4.50	1.33	3.74	1.89	−1.72	0.48
RF-Self	3.52	1.04	3.60	1.17	3.41	0.85	−0.65	0.18
RF-Others	4.13	1.38	4.16	1.14	4.11	1.67	−0.12	0.04
Mothers’ RF								
RF-Global	4.17	1.90	4.17	1.87	4.17	2.00	0.00	0.00
Youths’ symptoms								
SRP–Anxiety^a^	56.57	11.18	61.33	11.67	51.36	8.02	−3.35^*^	0.97
SRP–Internalizing^a^	52.24	10.35	54.79	11.19	49.45	8.77	−1.79	0.52
PRS–Anxiety^a^	58.60	13.41	66.50	11.01	50.33	10.49	−4.93^**^	1.50
PRS–Internalizing^a^	62.19	16.65	73.27	14.10	50.57	9.83	−6.10^**^	1.83
Youths’ attachment								
CAI–Preoccupation–Idealization^b^	0.25	2.56	−0.09	2.80	0.73	2.27	1.07	0.32
CAI–Security–Dismissing^c^	11.68	9.28	12.05	8.15	11.24	10.68	−0.29	0.09
Mothers’ attachment								
AAI–Preoccupation	0.26	2.38	0.73	2.57	−0.39	1.96	−1.68	0.48
AAI–Dismissing	−0.43	5.81	0.24	5.82	−1.29	5.82	−0.90	0.26

RF=reflective functioning; SRP=self-report of personality; PRS=parent rating scale; CAI=child attachment interview; AAI=adult attachment interview.

a Standardized (*t*) scores.

b Higher score=lower preoccupation and higher idealization.

c Higher score=higher security and lower dismissal.

* *p*<0.05;

** *p*<0.01.

Regarding symptoms, youths in the clinical group had higher scores of anxiety symptoms (self- and mother-reported). However, they did not differ from non-anxious youths in their level of self-reported internalizing symptoms.

Zero-order correlations between all study variables in the overall sample (Table 2) revealed that gender was the only sociodemographic variable related to outcome variables (i.e., anxiety or internalizing symptoms). Therefore, regression analyses were conducted both with and without controlling for gender. Youths’ RF-Self was the only RF variable correlated with symptoms. Nevertheless, youths’ RF-Others was also entered as an independent variable in the regression models to meet the study’s objectives.

**Table 2:** Zero-order correlations between study variables in the overall sample (*N*=53).

S. No.		1	2	3	4	5	6	7	8	9	10	11	12	13	14	15	16	17
1.	Group	-																
2.	Gender (male)	−0.29^*^	-															
3.	Age	−0.02	0.03	-														
4.	Family income	0.06	0.04	−0.21	-													
5.	Mothers’ education	−0.26	0.02	−0.15	0.36^*^	-												
6.	RF-Global	0.23	−0.39^**^	0.14	0.19	0.12	-											
7.	RF-Self	0.09	−0.46^**^	0.12	0.00	0.10	0.58^**^	-										
8.	RF-Others	0.02	−0.28^**^	0.32^*^	0.14	0.20	0.85^**^	0.55^**^	-									
9.	Mothers’ RF	0.00	−0.05	−0.04	0.07	0.21	0.19	0.23	0.18	-								
10.	SRP–Anxiety^a^	0.45^**^	−0.38^*^	0.12	−0.10	−0.00	0.18	0.27	0.25	−0.07	-							
11.	SRP–Internalizing^a^	0.26	−0.25	0.16	−0.40	−0.01	0.09	0.37^*^	0.21	0.05	0.75^**^	-						
12.	PRS–Anxiety^a^	0.61^**^	−0.28	0.10	−0.20	−0.29	0.10	0.05	−0.08	−0.18	0.25	0.19	-					
13.	PRS–Internalizing^a^	0.69^**^	−0.34^*^	0.12	−0.19	−0.16	0.18	0.19	−0.05	−0.14	0.40^**^	0.40^**^	0.84^**^	-				
14.	CAI–Preoc.–Ideal^b^	−0.16	0.29^*^	0.04	−0.23	−0.08	−0.45^**^	−0.24	−0.38^**^	−0.05	−0.00	−0.04	−0.04	−0.06	-			
15.	CAI–Secu.–Dismis^c^	0.04	−0.32^*^	−0.06	0.17	0.09	0.78^**^	0.57^**^	0.70^**^	0.11	0.15	0.18	0.05	0.13	−0.66^**^	-		
16.	AAI–Preoccupation	0.24	−0.33^*^	0.07	−0.18	−0.18	0.11	−0.02	0.10	0.01	0.33^*^	0.31^*^	−0.06	0.13	−0.31^*^	0.10	-	
17.	AAI–Dismissing	0.13	−0.15	0.10	0.01	0.08	0.08	0.19	0.06	−0.18	0.29	0.15	−0.24	0.02	0.08	−0.14	0.35^*^	-

RF=reflective functioning; SRP=self-report of personality; PRS=parent rating scale; CAI=child attachment interview; AAI=adult attachment interview.

a Standardized (*t*) scores.

b Higher score=lower preoccupation and higher idealization.

c Higher score=higher security and lower dismissal.

* *p*<0.05;

** *p*<0.01.

As expected, youths’ attachment was associated with their RF capacities (Table 2). Specifically, youths’ attachment security was strongly and positively correlated with their global RF, RF-Self, and RF-Others scores. Conversely, youths’ idealization score was negatively correlated with their global RF and RF-Others scores. Finally, maternal attachment preoccupation was the only attachment variable significantly correlated with youths’ anxiety and internalizing symptoms. Therefore, maternal attachment preoccupation, but not maternal dismissal nor youths’ attachment, was included as a covariate in the regression analyses.

### Predictors of Youths’ Anxiety and Internalizing Symptoms

Because RF was associated with self-reported – but not mother-reported – anxiety and internalizing problems (Table 2), regressions were performed to predict these specific outcomes. As a first step, multiple linear regressions were conducted to test the predictive effect of RF-Self and RF-Others on anxiety and internalizing symptoms without controlling for attachment or gender. RF predicted self-reported internalizing symptoms, but not anxiety (Table 3). Specifically, a higher score of RF-Self predicted a higher level of self-reported internalizing symptoms. Together, RF-Self and RF-Others explained 10% of the variance in internalizing symptoms (Cohen’s *f*^2^=0.11; small effect size).

**Table 3:** Multiple linear regression models predicting youths’ self-reported symptoms (overall sample; *N*=53).

	*Adjusted R* ^2^	*F*	*p*	*B (SE)*	*β*	*t*	*p*	[95% CI]
DV: Self-reported anxiety symptoms	0.04	2.02	0.146					
RF-Self				2.06 (1.89)	0.19	1.09	0.282	[−1.75–5.87]
RF-Others				1.07 (1.37)	0.14	0.79	0.437	[−1.69–3.84]
DV: Self-reported internalizing symptoms	0.10	3.49	0.039					
RF-Self				3.75 (1.70)	0.38	2.21	0.033	[0.32–7.17]
RF-Others				−0.04 (1.23)	−0.01	−0.03	0.973	[−2.52–2.44]

DV=dependent variable; SE=standard error; CI=confidence interval; RF=reflective functioning.

Secondly, sequential regressions analyses were conducted to investigate whether RF predicted youths’ symptoms beyond the effect of attachment (Table 4). Maternal attachment preoccupation significantly predicted self-reported symptoms of anxiety and of internalization. Youths’ higher RF-Self significantly predicted higher self-reported internalizing symptoms after controlling for attachment. Youths’ RF accounted for an additional 14.7% of the internalizing symptoms’ variance, beyond maternal attachment preoccupation.

**Table 4:** Sequential regression models predicting youths’ self-reported symptoms, controlling for mothers’ attachment (overall sample; N=53).

	Self-reported anxiety symptoms					Self-reported internalizing symptoms				
Predictor	*ΔR* ^2^	*ΔF*	*df*	B [95% CI]	*ß*	*ΔR* ^2^	*ΔF*	*df*	B [95% CI]	*ß*
Block 1	0.11	5.02^*^	1, 41			0.10	4.49^*^	1, 41		
AAI–Preoccupation				1.50 [0.15, 2.85]	0.33^*^				1.31 [0.06, 2.57]	0.31^*^
Block 2	0.08	1.81	2, 39			0.15	3.81^*^	2, 39		
RF-Self				2.25 [−1.70, 6.20]	0.21				4.31 [0.81, 7.82]	0.43^*^
RF-Others				0.79 [−2.03, 3,61]	0.10				−0.58 [−3.08, 1.92]	−0.08

AAI=adult attachment interview; RF=reflective functioning; df=degrees of freedom; CI=confidence interval.

* *p*<0.05.

RF-Self remained marginally predictive (*p*=0.055) of self-reported internalizing symptoms, after controlling for both attachment and gender (Table 5).

**Table 5:** Sequential regression models predicting youths’ self-reported symptoms, controlling for mothers’ attachment and youngsters’ gender (overall sample; *N*=53).

	Self-reported anxiety symptoms					Self-reported internalizing symptoms				
Predictors	*ΔR* ^2^	*ΔF*	*df*	B [95% CI]	*ß*	*ΔR* ^2^	*ΔF*	*df*	B [95% CI]	*ß*
Block 1	0.13	6.32^*^	1, 41			0.07	2.95	1, 41		
Male gender				−8.34 [−15.05, −1.64]	−0.37^*^				−5.45 [−11.87, 0.96]	−0.26
Block 2	0.05	2.33	1, 40			0.06	2.64	1, 40		
AAI–preoccupation				1.05 [−0.34, 2.45]	0.23				1.07 [−0.26, 2.40]	0.26
Block 3	0.02	0.58	2, 38			0.12	3.13^†^	2, 38		
RF-Self				1,28 [−3.14, 5.69]	0.12				4.63 [0.66, 8.59]	0.46^*^
RF-Others				0.70 [−2.12, 3.53]	0.09				−0.55 [−3.09, 1.98]	−0.08

AAI=Adult Attachment Interview; RF=reflective functioning; df=degrees of freedom; CI=confidence interval.

* *p*<0.05;

† *p*=0.055.

Finally, further analyses were performed to assess the specific associations between RF and the Depression subscale of the internalizing symptoms scale. Data for the Somatization subscale was available for only 19 participants because it is solely included in the 12–21-year-old version of the BASC-2 SRP. Therefore, further analyses could not be performed for this subscale. Zero-order correlation showed a moderate positive association between RF-Self and the Depression subscale (*r*=0.31, *p*=0.039). The Depression subscale’s scores were not associated with any other types of RF.

## Discussion

This study’s objective was to assess the relative contributions of mothers’ and youths’ RF to children’s and adolescents’ anxiety diagnosis and symptoms, and to internalizing problems more broadly. Specifically, the study aimed to assess the contributions of specific dimensions of youth’s RF (general, regarding self, or regarding others) to anxiety and the broader internalizing difficulties spectrum. We hypothesized that the presence of an anxiety disorder and a higher level of internalizing symptoms (anxiety and depression) would be predicted by lower mothers’ RF, and by lower youths’ global and self-related RF. Results partially confirmed our hypotheses. Despite the theoretical literature suggesting a negative association between mentalization and psychopathology, our results showed no difference in the RF abilities of clinically anxious and non-anxious youths, and an unexpected, positive, association between self-related RF and internalizing difficulties. In that, they complement a certain body of empirical studies, as discussed below.

### Specific Contributions of Youths’ RF Dimensions

Unexpectedly, the only association of RF with symptoms was found between youths’ RF-Self and internalizing difficulties. Indeed, youths with better RF capacities regarding self reported more internalizing symptoms, even after controlling for mother’s attachment. This effect remained marginally significant after controlling for both maternal attachment and youth’s gender.

The association between good RF-Self and higher internalizing symptoms could be understood in the light of the “self-absorption paradox” (Trapnell and Campbell, 1999). According to Benbassat and Priel (2012), who found a similar pattern between paternal RF and adolescents’ internalizing problems, high RF capacities would increase self-consciousness and the accuracy of self-perception, for better and for worse. While being able to more accurately reflect upon oneself, youths with good RF capacities would also be more conscious of their difficulties and of less desirable aspects of themselves, thus more prone to report emotional or behavioral difficulties (Benbassat and Priel, 2012; Benbassat and Shulman, 2016). This hypothesis is coherent with our finding that RF-Self was associated with youths’ self-reported – but not parent-reported – internalizing symptoms.

Further analyses revealed that among the subscales of internalizing problems on the BASC-2, only Depression was positively associated with RF-Self, whereas Anxiety was not. Thus, a high capacity to reflect upon oneself seems to be more strongly related to the depressive – as opposed to the anxious – facet of internalizing difficulties. A similar pattern was found among substance-abusing mothers. Mothers’ RF-Self, assessed with the Parent Development Interview, was positively associated with their depressive symptoms (Borelli et al., 2012). This suggests that the “self-absorption paradox” might be more prominent when participants are already prone to self-focused rumination as is the case with depression (Luyten et al., 2012). Studies in the language field have also raised the idea that first-person discourse speech (“I-talk”) would be associated with enhanced negative emotionality and thus be a marker of depressive symptomatology (e.g., Tackman et al., 2019). Further studies are, however, required to provide a deeper understanding of language and RF in the distinction between anxious and depressive psychopathology.

Secondly, contrary to our hypothesis, youths’ general RF was not associated with the presence of an anxiety disorder neither with anxiety symptoms. Although unexpected, these results appear to be in line with those from studies among non-clinical samples. Indeed, the absence of relation between mentalization and anxiety symptoms in healthy children and adolescents from the general population has been found elsewhere (e.g., Neath et al., 2013; Steenhuis et al., 2019). This might suggest that our clinical sample resembles the normative population, whether indicating a selection bias or simply that participating families of anxious youths shared common characteristics with those of non-anxious. In fact, apart from reported symptoms of anxiety and internalizing difficulties, the clinically anxious and non-anxious groups were alike on all other outcomes. Another reason for the absence of association between RF and anxiety in the present study might be because anxiety was considered as a global rather than a multidimensional construct. Indeed, different types of anxiety (e.g., specific vs. social phobia) may show different patterns of association with mentalization. For instance, mentalization was inversely associated with symptoms of separation anxiety and panic disorder, but not with generalized anxiety, among school-aged children of the general population (Caputi and Schoenborn, 2018). Unfortunately, the present study’s sample size prevented us from testing this hypothesis.

Finally, we must address the possibility that high RF-Self could be an artifact of hypermentalization, which is an excessive and inaccurate interpretation of behavioral cues and mental states in oneself or others (“*too much of a good thing*”; Sharp and Venta, 2012). Hypermentalization can be mistaken for good mentalizing, thereby artificially inflating RF scores (Chow et al., 2017). However, the RF coding instrument used in our study, the CARFS, has specific coding rules for hypermentalizing passages (where the participant’s response sounds “canned” or over-analytical). Such responses cannot receive scores higher than 3 or 4 on the 9-point RF scale. In our global sample, RF-Self scores range from 1 to 5.5, so the highest scores correspond to genuinely good mentalization. We therefore suggest that the association between higher youths’ RF-Self and higher internalizing symptoms found in this study is not explained by hypermentalization.

### Mothers’ Attachment and RF and Youths’ Symptoms

Contrary to previous studies suggesting that low mothers’ RF would put children at risk of emotional difficulties (Esbjørn et al., 2012, 2013; Dubois-Comtois et al., 2019), we found no association between mothers’ RF and youths’ anxiety (disorder or symptoms) and internalizing difficulties more broadly. The older age of youths in our sample might lessen this association. Parents’ RF is thought to be determinant in the emotional co-regulation process within the attachment relationship in the early years (Fonagy and Target, 2005). As children grow older, they acquire emotional and cognitive abilities such as abstract thinking, making them better at understanding, regulating, and reflecting on their own and others’ mental states (Fonagy et al., 2002; Chow et al., 2017). RF is expected to be well developed by the age of 7 or 8years old and to become more sophisticated during early adolescence (Midgley et al., 2017). Thus, as they age, children’s psychological adjustment would be less related to their parents’ RF abilities than to their own. In line with this idea of a reduced impact of parental RF as children age, there was no association between mothers’ RF and youths’ attachment security in the present study.

Maternal attachment preoccupation, but not dismissal, was associated with youths’ self-reported anxiety and internalizing symptoms. Attachment preoccupation is characterized by hyperactivation strategies in the attachment relationship, that is, an amplification of the distress expression and an excessive search for reassurance from the attachment figure. Mothers’ high preoccupation with their own attachment figures is likely to lessen their sensibility to their child (i.e., how they perceive their child’s attachment signals and how they respond to it) and thus influence their parental practices (van IJzendoorn, 1995). They are therefore more likely to provide excessive or intrusive care to their children, which in turn limits children’s development of autonomy and enhances anxiety. Nevertheless, youths’ RF-Self added a significant contribution to their symptoms beyond the influence of mothers’ attachment preoccupation, highlighting the importance of considering RF when studying the influence of parents’ attachment on older children and adolescents’ psychological adjustment.

### Differences Between Anxious and Non-anxious Children and Adolescents

Although RF-Self was positively associated with internalizing symptoms in the overall sample, it failed to differentiate the clinical from the non-clinical group. Indeed, youths with an anxiety disorder in our sample are as good as their non-anxious peers in reflecting upon their own and others’ mental states. This finding is in line with those of Breinholst et al. (2018) who found that although clinically anxious school-aged children had lower relationship-triggered RF, they were as good as their non-anxious counterparts in non-social RF (“developmental perspective”). Thus, the very nature of the questions (anxiety triggering or not) used to assess RF might contribute to between-study variations in RF among anxious children. In our study, youths’ RF was assessed *via* a task that triggers the attachment system (CAI). Although the CAI taps relationship representations, this might not be the prime anxiety-trigger for our clinical group in which the most prevalent anxiety disorder was specific phobia (46.7%), followed by generalized anxiety (33.3%). Anxiety disorders specific to relational contexts, namely separation anxiety and social phobia, were less prevalent (respectively, 26.7 and 10%). The assessment of RF in the context of an attachment interview might not have been a strong trigger for children and adolescents in our study, given the prominence of non-relational anxiety disorders in the sample. This also suggests that RF difficulties among clinically anxious individuals would be specific to certain tasks or contexts and intrinsically related to the nature of their anxieties. In that, our results support the relevance of the corpus of studies focusing on symptom-specific RF in clinical populations (e.g., Kullgard et al., 2013; Keefe et al., 2019; Solomonov et al., 2019). Symptom-specific RF refers to the capacity to reflect on the psychological roots of anxious – or any other pathological – manifestations (e.g., Why do you think you have panic attacks?; Rudden, 2017). For example, symptom-specific RF of clinically anxious adult patients was found to be significantly lower than their general RF capacities (Rudden et al., 2008; Kullgard et al., 2013). Those studies are of particular importance to understand how therapeutic processes can best help reduce symptoms. However, symptom-specific RF remains to be studied among children and adolescents. For instance, future studies assessing RF with the CAI could add questions that prompt reflection on symptoms specific to the youths’ clinical condition.

Furthermore, generalized anxiety, which was the second diagnosis in importance in our clinical sample, may even provide a favorable ground for the development of RF capacities. Whereas socially anxious children tend to fear, misinterpret, or avoid social contexts, generally anxious individuals tend to grasp their overall environment as potentially dangerous. Hypervigilance and apprehensive expectation are common traits of generally anxious individuals (American Psychiatric Association, 2013). If anything, such apprehensions might prove an advantage for RF development. While being constantly alert to potential threats in their relational and physical environments, anxious youths deploy a lot of energy to anticipate the behaviors and mental states of others. This “reflective training” might prevent clinically anxious children to stand out in terms of RF difficulties, especially in understanding others’ behavior.

The positive association between RF and symptoms could also suggest that despite their fairly good mentalizing capacities, emotion regulation remains difficult for anxious children and adolescents. In that, the *cognitive* vs. *affective* dimension of mentalization (Fonagy and Bateman, 2019) could be useful in understanding the dynamics of anxious individuals’ RF. We could hypothesize that their cognitive strategies with regard to potential threats would make them good in the *cognitive* dimension of mentalization (i.e., the capacity to name and think about mental states), whereas the *affective* dimension (i.e., the capacity to appreciate the emotional component of mental states) would be less developed or perhaps inhibited by the anxious state of arousal. In other words, it might be easier for an anxious youth to rationalize emotional states than to truly regulate them. Therefore, RF studies among clinical populations would benefit from assessing thoroughly the mentalizing profile (Luyten et al., 2019) of participants to elicit their strengths and weaknesses on each of the dimensions of mentalization (automatic vs. controlled; self-oriented vs. others-oriented; internal vs. external; cognitive vs. affective) to further specify the therapeutic interventions to be favored.

### Limitations and Future Directions

This study has limitations, which must be acknowledged. Mainly, the relatively small size of our sample limits the scope of possible analyses due to reduced statistical power. This sample size (*N*=53) nonetheless allowed for detecting large effects when running multiple regression analyses with two to four predictors (Cohen, 1992). Also, our clinical sample included children and adolescents with heterogeneous and comorbid anxiety diagnoses, thus preventing us from assessing the specific associations between RF and each type of anxiety. Because the association between mentalization and mental health is multidimensional, future studies should aim at assessing the associations between each dimension of mentalization and specific disorders. The uneven distribution of boys and girls in the two groups should also be pointed out. Although the effect of RF-Self on internalizing symptoms remained marginally significant after controlling for gender, our results showed an intriguing pattern suggesting lower RF capacities in boys. Our sample was too small to conduct moderation analyses, but it would be enlightening to do so in future research to outline the role of gender in the relation between RF and psychological adjustment. Moreover, our results are limited by the fact that we used a single, attachment-related instrument to assess RF. It is reasonable to assume that anxious youths have more difficulties mentalizing about themselves when asked to think about their anxiety symptoms than when questioned about the relation with their attachment figures, especially in the case of secure attachment. As discussed previously, by coupling the RF scale in attachment interviews with an RF instrument that specifically targets anxiety, we would gain an even more acute understanding of RF in relation to psychopathology. Another, simpler, way of doing so would be to add a question at the end of the CAI and AAI asking how the participant reflects on his/her symptoms. Finally, our study is limited by the sole inclusion of mothers. Thus, potentially relevant information is lost regarding the child’s exposition to parental mentalization and the influence of the intergenerational transmission of RF on mental health. Studies that include fathers’, in addition to mothers’, RF would be of great interest, especially during adolescence. Indeed, during this developmental period, the father–child relation is thought to be particularly significant, notably in the separation–individuation process (Shulman and Seiffge-Krenke, 1997). One study found an intriguing positive association between fathers’ – but not mothers’ – RF and adolescents’ internalizing symptoms (Benbassat and Priel, 2012; Benbassat and Shulman, 2016). The authors emphasized the impact of fathers’ outcomes in the psychological adjustment of their teenagers. More studies are needed on the association between RF of both parents and youths’ anxiety and internalizing symptoms before these impacts could be better understood.

### Clinical Implications

Our findings have interesting implications for clinicians working with anxious youths and their families. First, the positive association between RF-Self and internalizing symptoms highlights how an increased ability to reflect on one’s own mental states can inform the clinician about the possibility of a more depressive – as opposed to anxious – component of the internalizing symptomatology. As noted previously, more studies are needed to support and demystify this preliminary finding. Nevertheless, considering the high rate of comorbidity between anxiety and depressive disorders, a thorough assessment of the patient’s RF capacities can be a useful tool for the clinician in clarifying the internalizing symptomatology and consequently determine the most appropriate intervention.

Secondly, despite a corpus of studies linking mentalization deficits to psychopathology, our results suggest that such an association is not as obvious when it comes to anxiety. In the light of our conclusions, clinicians should keep in mind that their young anxious patients as well as their parents might be fairly good in thinking about and making sense of their feelings and internal states. This appears to especially be the case in situations where the patient’s fears are not specifically triggered. Moreover, it seems like the mentalization capacities of anxious youths, despite being adequate, fail to help them self-regulate. Therefore, a thorough assessment of how the patient uses his/her RF skills is crucial. Such assessment should thus go beyond establishing the level of mentalization abilities to evaluate the impact of these abilities on the patient’s mental health. For instance, clinicians should seek to answer to questions such as “Do RF-S abilities help the patient to self-regulate or, rather, exacerbate self-consciousness and negative rumination?” and “How does the patient use his/her RF abilities under stressful conditions?” Therapeutic interventions such as mentalization-based treatments (MBT) could be particularly helpful in promoting emotional regulation strategies that could be used alongside reflective capacities (Midgley et al., 2017; Achim et al., 2020b). Indeed, while being careful that the patient’s self-consciousness does not enhance his/her emotional distress (*via* the so-called self-absorption paradox), clinicians should aim at helping young patients use their RF skills in anxiety-provoking situations to gain better emotional regulation. For example, with cognitive-behavioral interventions such as gradual exposure to the object of fear, the clinician can stimulate the patient’s RF regarding his/her internal states when confronted with the anxiety-provoking situation. Questions such as “Can you describe how you were feeling during the exposure?,” “Do you notice any changes in your feelings since the last level of exposure?,” and “What links can you make between your bodily sensations and your emotions?” require a good RF capacity and are central in the therapeutic process of desensitization. Similarly, for patients with rather relational anxiety such as social phobia, the therapy itself is likely anxiety-provoking. Thus, working on RF capacities within the therapeutic relationship could help patients develop insights about their thoughts and feelings when exposed to interpersonal situations and develop better regulation strategies as they learn to tolerate internal states that were previously uncomfortable. This is in line with research findings among patients with panic disorder revealing that higher emotional expression in patients during therapy leads to a greater reduction of symptoms (Keefe et al., 2019). In summary, the present study suggests that clinical work with anxious youths should go beyond fostering mentalizing abilities to support and promote the development of emotion regulation strategies and resiliency, which are usual components of MBT (Midgley et al., 2017; Achim et al., 2020b).

## Data Availability Statement

The raw data supporting the conclusions of this article will be made available by the authors, without undue reservation.

## Ethics Statement

This study received full approval from the scientific and ethical boards of the University of Sherbrooke and Sainte-Justine University Hospital (Quebec, Canada). Written informed consent to participate in this study was provided by the participants’ legal guardian/next of kin.

## Author Contributions

VC contributed to the data analysis and wrote the first draft of this manuscript as a part of her doctoral thesis. VS was responsible for designing the larger research project from which this study stems and supervised the data analysis and the manuscript writing. JA contributed to the conceptualization of the study and to revising the manuscript. PB and TB-T contributed to the data coding and analysis and revising the manuscript. All authors contributed to the article and approved the submitted version.

## Funding

This research was supported by grants from the Fonds de recherche du Québec – Société et culture (FRQSC: 164671) and the Social Sciences and Humanities Research Council (SSHRC 430-2012-0417) of Canada.

## Conflict of Interest

The authors declare that the research was conducted in the absence of any commercial or financial relationships that could be construed as a potential conflict of interest.

## Publisher’s Note

All claims expressed in this article are solely those of the authors and do not necessarily represent those of their affiliated organizations, or those of the publisher, the editors and the reviewers. Any product that may be evaluated in this article, or claim that may be made by its manufacturer, is not guaranteed or endorsed by the publisher.
